# Methylene Blue and Blood Transfusion in Hemorrhagic Shock Resuscitation: An Experimental Porcine Study

**DOI:** 10.21470/1678-9741-2023-0480

**Published:** 2024-10-14

**Authors:** André Luppi, Agnes Afrodite S. Albuquerque, Marelaine Prandi, Jessyca M. Barbosa, Maria Cecília Jordani, Suely Fazio Ferraciolli, Sergio Wechsler, Paulo Roberto B. Evora

**Affiliations:** 1 Department of Surgery and Anatomy, Faculdade de Medicina de Ribeirão Preto, Universidade de São Paulo, Ribeirão Preto, São Paulo, Brazil; 2 Department of Radiology, Faculdade de Medicina da Universidade de São Paulo, Universidade de São Paulo, Ribeirão Preto, São Paulo, Brazil; 3 Deparment of Statistics, Instituto de Matemática e Estatística, Universidade de São Paulo, São Paulo, São Paulo, Brazil

**Keywords:** Animal Model, Methylene Blue, Hemorrhagic Shock, Circulatory Shock, Bleeding Control, Blood Transfusion.

## Abstract

**Introduction:**

Hemorrhagic shock requires immediate treatment to prevent mortality and organ dysfunction. This study evaluates the efficacy of methylene blue (MB) with blood transfusion (BT) as a potential rescue therapy in acute severe bleeding in pigs.

**Methods:**

Thirty animals were randomly assigned to one of six groups following the induction of fixed-pressure hemorrhagic shock, after reaching a mean arterial pressure (MAP) of 55 mmHg - Group 1 (60 BT: BT after 60 minutes), Group 2 (60 MB: MB infusion after 60 minutes), Group 3 (60 MB + BT: MB and BT after 60 minutes), Group 4 (15 MB + BT: MB and BT after 15 minutes), Group 5 (15 BT + 60 MB: BT after 15 minutes and MB infusion after 60 minutes), and Group 6 (15 MB + 60 BT: MB infusion after 15 minutes and BT after 60 minutes). Hemodynamic and blood gas parameters were meticulously recorded, reversal of the shock was considered when MAP reached 90% of the baseline MAP.

**Results:**

Except for Group 2, all groups reverted from the shock. However, groups that received MB in combination with BT, specifically Groups 3 and 4, exhibited statistically significant higher ratios of maximum MAP to baseline MAP.

**Conclusion:**

Using MB concomitant with BT allowed the reversal of hemorrhagic shock with higher median arterial pressure levels compared to BT alone or applying MB separately from BT. This suggests that simultaneous application of MB and BT could be a more effective strategy for reversing the effects of severe acute bleeding.

## INTRODUCTION

**Table:** 

Abbreviations, Acronyms & Symbols				
BT	= Blood transfusion		MDA	= Malondialdehyde
cGMP	= Cyclic guanosine monophosphate		NO	= Nitric oxide
CI	= Cardiac index		NOx	= Plasma nitrite and nitrate
CO	= Cardiac output		PAP	= Pulmonary arterial pressur
CVP	= Central venous pressure		PCP	= Pulmonary capillary pressure
GMP/NO	= Guanosine monophosphate/nitric oxide		PVR	= Pulmonary vascular resistance
HB	= Hemoglobin		PVRI	= Pulmonary vascular resistance index
Ht	= Hematocrit		SD	= Standard deviation
iNOS	= Inducible nitric oxide synthase		SVR	= Systemic vascular resistance
MAP	= Mean arterial pressure		SVRI	= Systemic vascular resistance index
MB	= Methylene blue			

Hemorrhagic shock is a life-threatening condition due to the loss of a large amount of blood and consequent circulatory failure, hindering O2 supply for tissue to maintain tissue metabolic demand. The main cause of hemorrhagic shock is trauma, and hemorrhagic shock is responsible for 30-40% of deaths in trauma victims. Other causes include surgical bleedings and rupture of the heart and great vessels^[1]^.

Hemorrhagic shock is associated with poor prognosis due to a complex chain of factors. The body responds to the bleeding with compensatory mechanisms to maintain homeostasis. Persisting the blood loss, compensatory mechanisms exhaust and lead to refractory shock. Inflammatory response starts right after the causative event and is aggravated by shock status, resuscitation, and hypothermia, being responsible for increasing oxidative stress, multiple organs disfunction, and death. The search for strategies that prevent multiple organ dysfunction and death even before hemostasis and the search for drugs that aid in treating hemorrhagic shock are significant challenges^[1]^.

Despite significant efforts to improve outcomes in the treatment of severe hemorrhage in recent years, experimental studies still need to propose new treatment strategies. Therefore, animal experiments are required to investigate the pathophysiology of hemorrhagic shock and identify new therapeutic approaches^[2]^.

Inflammation induces inducible nitric oxide synthase (iNOS), increasing the concentration of nitric oxide (NO) in quantities much higher than its physiological concentration, promoting vasodilatation observed in latter stages of shock. This effect of NO is a result of its role in signaling in cyclic guanosine monophosphate (cGMP) pathway^[3]^.

In an experimental model of refractory hemorrhagic shock in rabbits, the best hemodynamic response was achieved by volume resuscitation and its association with pharmacological inhibition of NO synthesis, suggesting that the NO pathway may be a strategy for treating this type of shock. The formation of NO and peroxide nitrate soon after the onset of hemorrhage precedes the expression of iNOS, expressed only in later phases after prolonged periods of shock^[3]^. This observation raises the hypothesis that blocking NO early, when endothelial constitutive NO synthase still produces NO, could lead to an adjuvant effect in reversing hemorrhagic shock.

Methylene blue (MB) is well known in the treatment of conditions like methemoglobinemia, and its safe profile is well established. It was found that MB is an inhibitor of guanylate cyclase, blocking cGMP production in the guanosine monophosphate/nitric oxide (GMP/NO) pathway. The drug has been widely studied in other types of shock, presenting advantages in pressure response, although its benefits in mortality needs more studies to be accessed^[3]^.

According to guidelines for treating hemorrhagic shock, an early approach is mandatory^[4]^. Although blocking the GMP/NO pathway is theoretically justifiable, few experimental studies or clinical trials have evaluated its use in treating hemorrhagic shock. No experimental study has evaluated early blockade of NO production with MB in this type of shock to date. The present study aimed to evaluate whether administering MB within the first 60 minutes of hemorrhagic shock in swine is safe and effective in resuscitating the shock state.

## METHODS

Male Dalland pigs (22-26 kg) were purchased from a specialized breeder. The Comissão de Ética no Uso de Animais (or Ethics Committee on Animal Experimentation) of Faculdade de Medicina de Ribeirão Preto, under protocol number 23/2015, approved all animal procedures and experimental protocols used in this study.

### Animal Preparation and Hemodynamic Parameters

The animals received preanesthetic intramuscular injection of xylazine (2 mg/kg) combined with ketamine (20 mg/kg) in the quadriceps muscle of one hind limb. After anesthetic induction, intravenous anesthesia was maintained (right jugular vein, central Swan-Ganz catheter) with midazolam (0.5 mg/kg/h) and fentanyl (3 µg/kg/h).

A 744HF75 Swan-Ganz CCOmbo CCO/SvO₂ catheter (Edwards Lifesciences, California, United States of America) was placed into the lumen of the pulmonary artery through the right jugular vein. The left carotid artery was catheterized to record the arterial pressure. The mean arterial pressure (MAP), pulmonary arterial pressure (PAP), pulmonary capillary pressure (PCP), and central venous pressure (CVP) were recorded using an MP System 100 A device (BioPac System Inc., California, United States of America). Additionally, cardiac output (CO), systemic vascular resistance (SVR), and pulmonary vascular resistance (PVR) were measured using a Vigilance System (Edwards Life Sciences LLC). For shock induction and laboratory sample collection, an arterial catheter was inserted into the right femoral artery.

Following instrumentation, hemodynamic stabilization was allowed for 20 minutes, and baseline values were recorded, as presented in Table 1. After that, animals were exsanguinated until they reached a MAP of 55 mmHg. The blood was collected in bags for later retransfusion. The average weight of the blood bags from the 30 animals was 645.04 g ± 185.3 g (mean ± standard deviation [SD]), and the time to reach the preconized pressure was 14.75 min ± 5.29 (mean ± SD).

**Table 1:** Baseline values of hemodynamic variables in each experimental group.

	Group 1	Group 2	Group 3	Group 4	Group 5	Group 6
	BT 60	MB 60	MB + BT 60	MB + BT 15	BT 15 + MB 60	MB 15 + BT 60
Baseline mean arterial pressure (mmHg)	116.5	115	105.5	105	107	110
Baseline pulmonary arterial pressure (mmHg)	30.5	31	29.5	32	29	32
Baseline pulmonary capillary pressure (mmHg)	22	21	22.5	21	20	22
Baseline central venous pressure (MmHg)	19	18	19.5	19	19	18
Baseline cardiac output (L/min)	2.7	2.9	2.1	2.8	2.6	3.5
Baseline cardiac index (L/min/m^2^)	1.6	1.8	1.4	1.8	1.3	2.1
Baseline systemic vascular resistance (Dyne-s/cm-5)	2786	2589	3550.5	2421	2898	2103
Baseline systemic vascular resistance index (Dyne-s/cm-5/m^2^)	4888	4142	5144.5	4061	5507	3994
Baseline pulmonary vascular resistance (Dyne-s/cm-5)	281	249	287.5	403	229.5	261
Baseline pulmonary vascular resistance index (Dyne-s/cm-5/m^2^)	449.5	422	427.5	617	482	532

BT=blood transfusion; MB=methylene blue

Upon reaching the target pressure, the chronometer was started (time = 0). Hemodynamic parameters were measured before the shock (baseline) and at 0, 10, 15, 20, 30, 40, 50, 60, 70, and 90 minutes, and laboratory measurements were also taken before the shock and at 0, 15, 30, and 90 minutes. Depending on their assigned group, the animals received the previously extracted amount of blood, MB, or a combination of both, at either 15 or 60 minutes post extraction. Reversal of the shock state was considered when MAP reached 90% of the baseline value. After the experiment, animals were humanely euthanized by exsanguination, still under deep sedation.

### Study Design

The animals (n = 30) were randomized and allocated to six groups after hemorrhagic shock induction: Group 1 (60 blood transfusion [BT]: blood retransfusion after 60 minutes), Group 2 (60 MB: MB infusion after 60 minutes), Group 3 (60 MB + BT: MB and BT after 60 minutes), Group 4 (15 MB + BT: MB and BT after 15 minutes), Group 5 (15 BT + 60 MB: BT after 15 minutes and MB infusion after 60 minutes), and Group 6 (15 MB + 60 BT: MB infusion after 15 minutes and BT after 60 minutes). Table 2 details the distribution of the animals among experimental groups.

**Table 2:** Distribution of the animals among the experimental groups.

Experimental Groups	Intervention
Group 1 (N=5^*^)	BT at 60 minutes
Group 2 (N=5)	MB at 60 minutes
Group 3 (N=5^*^)	MB + BT at 60 minutes
Group 4 (N=5)	MB + BT at 15 minutes
Group 5 (N=5)	BT at 15 minutes and MB at 60 minutes
Group 6 (N=5)	MB at 15 minutes and BT at 60 minutes

BT=blood transfusion; MB=methylene blue

^*^One animal from Group 1 and another from Group 3 experienced hemodynamic worsening and cardiac arrest prior to the intervention at the 60^th^ minute and were subsequently excluded from the experiment (N=4)

### Methylene Blue Administration

MB (1%) was prepared by mixing 10 mg MB powder with 1 ml of sterile water. A bolus dose of MB (2 mg/kg) was administered intravenously.

### Biochemical Control

Biochemical controls included serial measurements of arterial and venous blood gases, hemoglobin (HB), hematocrit (Ht), lactate, glycemia, urea, and creatinine, analyzed using a Gem Premier 3000 (Instrumentation Laboratory Co., Bedford, Massachusetts, United States of America).

### Plasma Nitrite and Nitrate

NO plasma concentrations of its stable end products, nitrite (NO2-) and nitrate (NO3-) are collectively known as NOx. After sampling, 1 ml of blood received 5 µl of heparin (1000 UI/ml). Samples were centrifuged for 10 minutes at 5,000 rpm, and the plasma was immediately separated, immersed in liquid nitrogen, and stored at -70°C. For the analysis, plasma deproteinization was performed with ethanol 95% at 4°C for 30 minutes and centrifugated at 10,000 rpm for five minutes. The resultant supernatant was submitted to NO/ozone chemiluminescence technique utilizing a NOAnalizer 280i (Sievers, Boulder, Colorado, United States of America). This concentration was then adjusted by a factor calculated from the quotient of the measured NOx and expected concentrations of sodium nitrate (5, 10, 25, 50, and 100 mmol), yielding a standard curve. The values are expressed in micrometers.

### Malondialdehyde

Malondialdehyde (MDA) is a final product of fatty acid peroxidation and is an indirect marker of oxidative stress. The MDA concentration was measured by spectrophotometry using the thiobarbituric acid technique. Blood samples were maintained on ice for further centrifugation at 4°C, 900 rpm for 20 minutes. A 50 µL sample of the supernatant plasma received 750 µL of phosphoric acid (0,44 mol/L) and 250 µL of thiobarbituric acid (42 mmol/L). Test tubes with the resultant solution were maintained in a bath at 100°C for 60 minutes, followed by a cold bath at 0°C. A volume of 0.5 ml of this solution received 0.5 ml of methanol-NaOH solution and was centrifugated at 9500 rpm for 5 minutes. 50 µL of the supernatant was submitted to the spectrophotometry at 532 nm Versamax (Molecular Devices, San Jose, California, United States of America). A standard curve with 1,1,3,3-tetramothoxypropane was used to calculate de MDA concentration.

### Statistical Analysis

Kruskal-Wallis nonparametric statistical test was used to compare the maximal/baseline ratio of studied parameters between groups. To assess differences between pairs of groups concerning their maximal/baseline MAP, it was used Wilcoxon two-tailed tests corrected using the Bonferroni scale. All analyses were conducted using Prism 5.0 (GraphPad Software Inc., San Diego, California, United States of America). Statistical significance was set at *P* < 0.05. A sample size of five per group provided 83% power with a 0.05 significance level to detect a relative effect > 20% between groups.

## RESULTS

During the experiment, one animal from Group 1 (60 BT) and one from Group 3 (60 MB + BT) experienced hemodynamic worsening and consequent cardiorespiratory arrest before the scheduled intervention at the 60^th^ minute. Consequently, they were excluded from the analysis in their respective groups.

### Mean Arterial Pressure

**Fig. 1:**
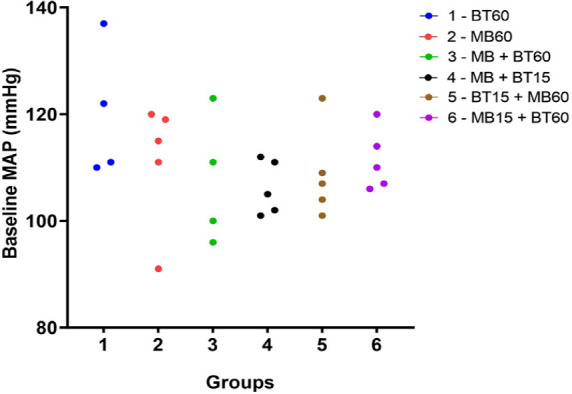
Baseline mean arterial pressure (MAP) of each animal in the different experimental groups. There was no statistical difference between the baseline MAP of the experimental groups. Blue (Group 1 60 BT), red (Group 2 60 MB), green (Group 3 60 MB + BT), black (Group 4 15 MB + BT), brown (Group 5 15 BT + 60 MB), and violet (Group 6 15 MB + 60 BT). Kruskal-Wallis nonparametric statistic = 4.573, with P > 0.45. BT=blood transfusion; MB=methylene blue.

There was no statistical difference between the baseline MAP of all experimental groups, confirming that the animals were adequately distributed among the six groups through randomization. These data are shown in Figure 1.

**Fig. 2:**
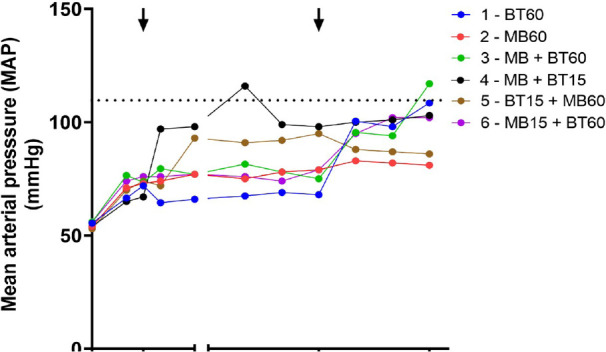
Time course of MAP values during the experiment. Arrows mark the intervention time for groups that received blood transfusion (BT) or methylene blue (MB). The dotted line represents de mean baseline values of MAP before the shock. Blue (Group 1 60 BT), red (Group 2 60 MB), green (Group 3 60 MB + BT), black (Group 4 15 MB + BT), brown (Group 5 15 BT + 60 MB), and violet (Group 6 15 MB + 60 BT).

**Fig. 3:**
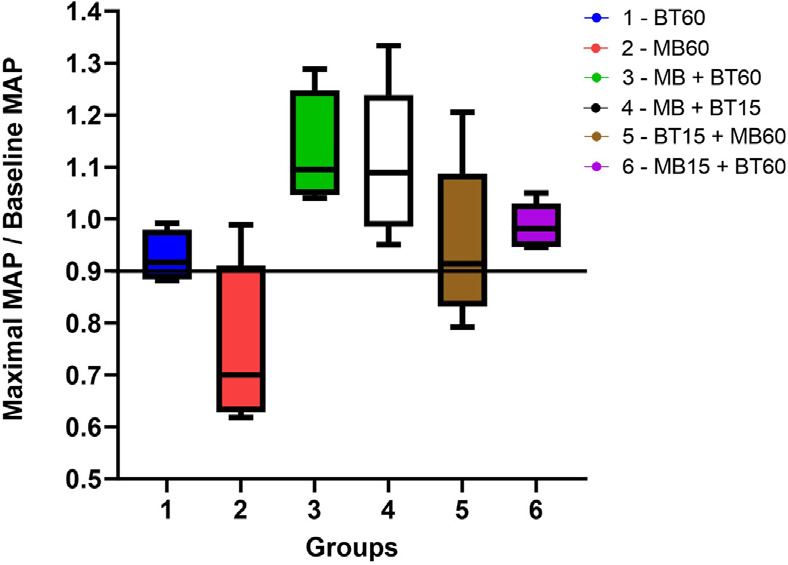
The ratio of maximum achieved pressure to baseline pressure between the experimental groups. Reversal of shock was considered when, after shock induction, the animal’s mean arterial pressure (MAP) reached > 90% of its baseline. The concomitant infusion of MB and blood led to a tendency for higher blood pressure levels compared to blood transfusion alone or the separate use of MB. MB alone did not lead to shock reversal. Group 1 (60 BT), Group 2 (60 MB), Group 3 (60 MB + BT), Group 4 (15 MB + BT), Group 5 (15 BT + 60 MB), and Group 6 (15 MB + 60 BT). Kruskal-Wallis nonparametric statistic = 15.914, with P = 0.007094 < 0.01. BT=blood transfusion; MB=methylene blue.

The time course of MAP is shown in Figure 2. To compare the experimental groups, the maximal MAP reached during the experiment was divided by the baseline MAP. Shock reversal was characterized by maximal MAP/baseline MAP > 90%. Except for Group 2, which received only MB, all other groups showed a reversal of shock. These data are shown in Figure 3. The highest maximum MAP/baseline MAP ratios were achieved in Groups 3, 4, and 6. A comparison among the six groups showed no statistical differences between Groups 3 and 4. However, Groups 3 and 6 and Groups 4 and 6 differed significantly. As for Groups 1 and 5, which also reverted from shock, their blood pressure values were borderline and did not present a statistical difference between them. The highest blood pressure responses were associated with the concomitant use of MB and BT, as evidenced by the responses of Groups 3 and 4. Paired statistical evaluations of the groups are presented in Table 3.

**Table 3:** Paired statistical evaluation of the blood pressure responses among the six experimental groups.

Paired Groups	Statistical Significance
1-2	0.009333333
1-3	0.001904667
1-4	0.002116667
1-5	0.06032^*^
1-6	0.007326667
2-3	0.001058
2-4	0.001058
2-5	0.010053333
2-6	0.003955333
3-4	0.06032^*^
3-5	0.007406667
3-6	0.002436667
4-5	0.010053333
4-6	0.009253333
5-6	0.009253333

Wilcoxon two-tailed tests with *P*-values corrected by Bonferroni scale and overall significance level of 5%. Bonferroni factor C (6.2) = 15.

* Statistically significant

### Pulmonary Arterial Pressure

**Fig. 4:**
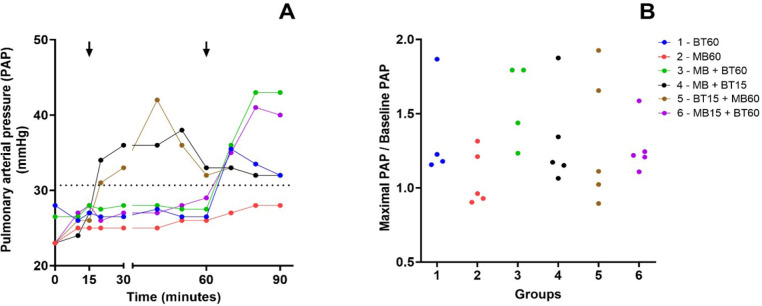
A) Time course of the median of PAP values during the experiment. Arrows mark the intervention time for groups that received blood transfusion (BT) or methylene blue (MB). The dotted line represents the mean baseline values of PAP before the shock. B) Relationship between the maximal PAP during the experiment and its baseline values among animals of each experimental group. There is no statistical difference between the groups. Blue (Group 1 - 60 BT), red (Group 2 - 60 MB), green (Group 3 - 60 MB + BT), black (Group 4 - 15 MB + BT), brown (Group 5 - 15 BT + 60 MB), and violet (Group 6 - 15 MB + 60 BT).

Figure 4A illustrates the evolution of PAP throughout the experiment. The relationship between the highest PAP value and the baseline value is shown in Figure 4B. There was no statistical difference in the increase in PAP concerning the baseline values between the groups; the results are shown in Figure 4.

### Pulmonary Capillary Pressure

**Supp. Fig. 1:**
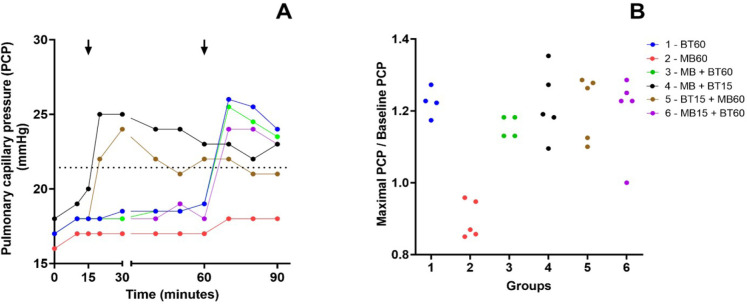
A) Time course of the median of pulmonary capillary pressure (PCP) values during the experiment. Arrows mark the intervention time in groups that received blood transfusion (BT) or methylene blue (MB). The dotted line represents de mean baseline values of PCP before the shock. B) Relationship between the maximal PCP during the experiment and its baseline value among animals of each experimental group. Group 2 presents lower PCP values compared to the other groups. Kruskal-Wallis nonparametric statistic = 13.57, with P = 0.0186 < 0.05. Blue (Group 1 - 60 BT), red (Group 2 - 60 MB), green (Group 3 - 60 MB + BT), black (Group 4 - 15 MB + BT), brown (Group 5 - 15 BT + 60 MB), and violet (Group 6 - 15 MB + 60 BT).

Except for Group 2 (60 MB), the other groups showed a similar increase in PCP related to BT. Data are presented in Supp. Figure 1.

### Central Venous Pressure

**Supp. Fig. 2:**
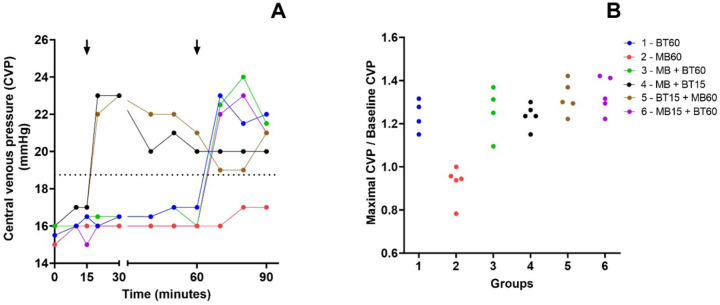
A) Time course of the median of central venous pressure (CVP) values during the experiment. Arrows mark the intervention time in groups that received blood transfusion (BT) or methylene blue (MB). The dotted line represents de mean baseline values of CVP before the shock. B) Relationship between the maximal CVP during the experiment and its baseline value among animals of each experimental group. Group 2 presents lower CVP values than the other groups. Kruskal-Wallis nonparametric statistic = 15.22, with P = 0.0094 < 0.01. Blue (Group 1 - 60 BT), red (Group 2 - 60 MB), green (Group 3 - 60 MB + BT), black (Group 4 - 15 MB + BT), brown (Group 5 - 15 BT + 60 MB), and violet (Group 6 - 15 MB + 60 BT).

Increased CVP occurred in all groups except the group that received only MB (Group 2). Related data are shown in Supp. Figure 2.

### Cardiac Output and Cardiac Index

**Supp. Fig. 3:**
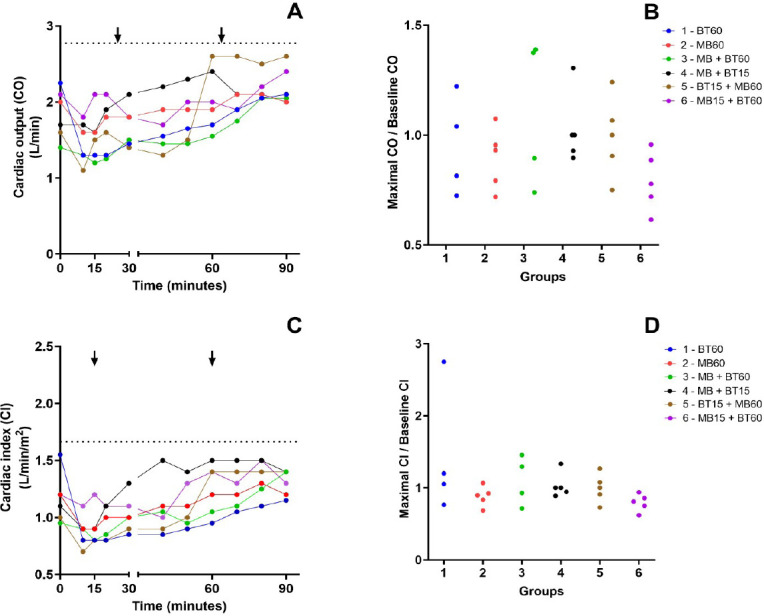
A) Time course of the median of cardiac output (CO) values during the experiment. Arrows mark the intervention time in groups that received blood transfusion (BT) or methylene blue (MB). The dotted line represents the mean baseline values of CO before the shock. B) Relationship between the maximal CO during the experiment and its baseline value among animals of each experimental group. There is no statistical difference between the groups. C) Time course of the median of cardiac index (CI) values during the experiment. Arrows mark the intervention time in groups that received BT or MB. The dotted line represents the mean baseline values of CI before the shock. D) Relationship between the maximal CI during the experiment and its baseline value among animals of each experimental group. There is no statistical difference between the groups. Blue (Group 1 - 60 BT), red (Group 2 - 60 MB), green (Group 3 - 60 MB + BT), black (Group 4 - 15 MB + BT), brown (Group 5 - 15 BT + 60 MB), and violet (Group 6 - 15 MB + 60 BT)

There were no variations in CO or cardiac index (CI) between the experimental groups. Data are presented in Supp. Figure 3.

### Vascular Resistances

**Supp. Fig. 4:**
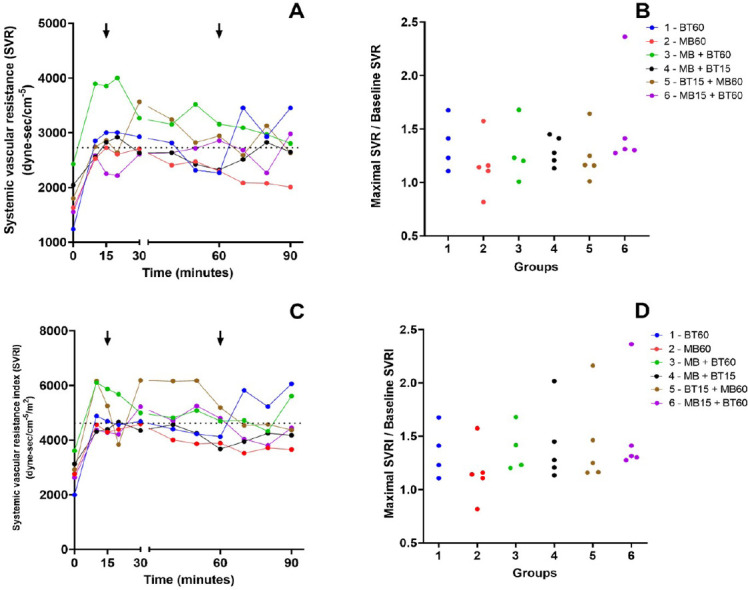
A) Time course of the median of systemic vascular resistance (SVR) values during the experiment. Arrows mark the intervention time in groups that received blood transfusion (BT) or methylene blue (MB). The dotted line represents the mean baseline values of SVR before the shock. B) Relationship between the maximal SVR during the experiment and its baseline value among animals of each experimental group. There is no statistical difference between the groups. C) Time course of the median of systemic vascular resistance index (SVRI) values during the experiment. Arrows mark the intervention time in groups that received BT or MB. The dotted line represents the mean baseline values of SVRI before the shock. D) Relationship between the maximal SVRI during the experiment and its baseline value among animals of each experimental group. There is no statistical difference between the groups. Blue (Group 1 - 60 BT), red (Group 2 - 60 MB), green (Group 3 - 60 MB + BT), black (Group 4 - 15 MB + BT), brown (Group 5 - 15 BT + 60 MB), and violet (Group 6 - 15 MB + 60 BT).

**Supp. Fig. 5:**
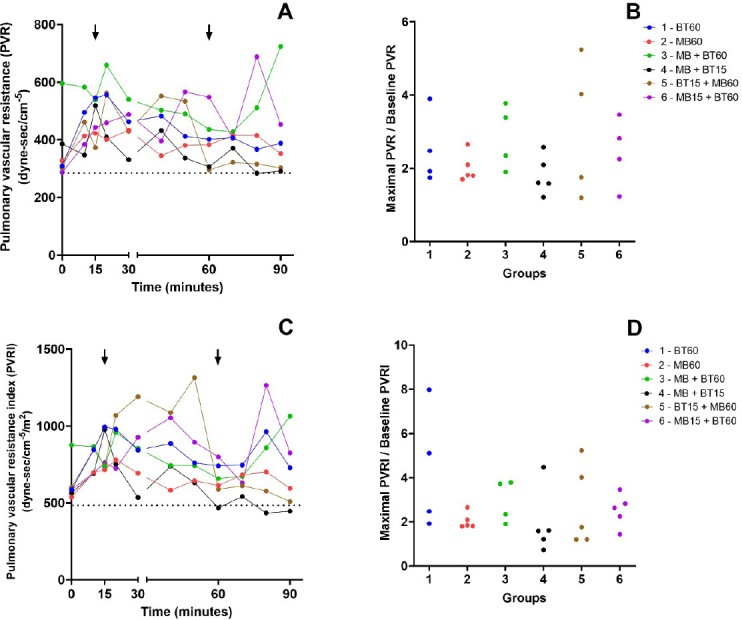
A) Time course of the median of pulmonary vascular resistance (PVR) values during the experiment. Arrows mark the intervention time in groups that received blood transfusion (BT) or methylene blue (MB). The dotted line represents the mean baseline values of PVR before the shock. B) Relationship between the maximal PVR during the experiment and its baseline value among animals of each experimental group. There is no statistical difference between the groups. C) Time course of the median of pulmonary vascular resistance index (PVRI) values during the experiment. Arrows mark the intervention time in groups that received BT or MB. The dotted line represents the mean baseline values of PVRI before the shock. D) Relationship between the maximal PVRI during the experiment and its baseline value among animals of each experimental group. There is no statistical difference between the groups. Blue (Group 1 - 60 BT), red (Group 2 - 60 MB), green (Group 3 - 60 MB + BT), black (Group 4 - 15 MB + BT), brown (Group 5 - 15 BT + 60 MB), and violet (Group 6 - 15 MB + 60 BT).

The evaluation of SVR, SVR index, PVR, and PVR index did not differ significantly between the groups. These data are shown in Supp. Figure 4 and Supp. Figure 5.

### Arterial Blood Acid-Base, Hemoglobin, Hematocrit, and Biochemical Analyzes

Arterial blood acid-base, HB, Ht, and biochemical analyses (sodium, potassium, glucose, and lactate levels) revealed no statistical differences between the experimental groups.

### Nitrate

**Supp. Fig. 6:**
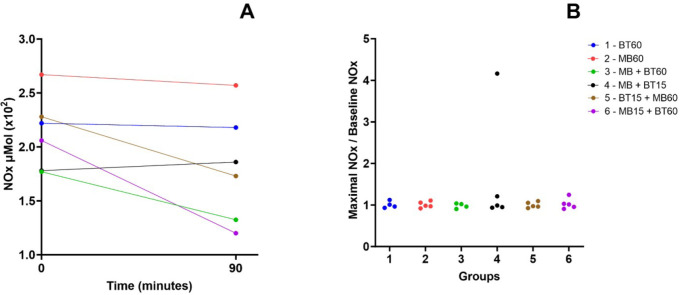
A). Initial and final plasma nitrite and nitrate (NOx) dosages values during the experiment. B) Relationship between the final dosage of NOx and its initial dosage among animals of each experimental group. There is no statistical difference between the groups. Blue (Group 1 - 60 BT), red (Group 2 - 60 MB), green (Group 3 - 60 MB + BT), black (Group 4 - 15 MB + BT), brown (Group 5 - 15 BT + 60 MB), and violet (Group 6 - 15 MB + 60 BT). BT=blood transfusion; MB=methylene blue.

There were no significant differences in nitrate levels between the groups. Data are presented in Supp. Figure 6 A.

### Malondialdehyde

**Supp. Fig. 7:**
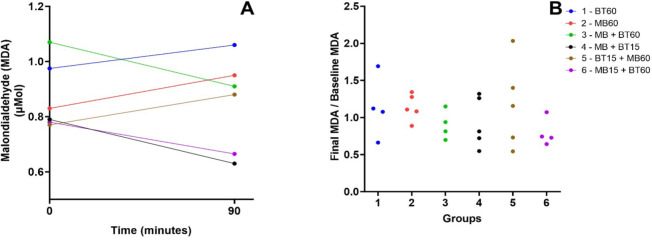
A) Initial and final malondialdehyde (MDA) dosages during the experiment. B) Relationship between the final dosage of MDA and its initial dosage among animals of each experimental group. There is no statistical difference between the groups. Blue (Group 1 - 60 BT), red (Group 2 - 60 MB), green (Group 3 - 60 MB + BT), black (Group 4 - 15 MB + BT), brown (Group 5 - 15 BT + 60 MB), and violet (Group 6 - 15 MB + 60 BT). BT=blood transfusion; MB=methylene blue.

No significant differences in MDA variations were observed between experimental groups. Data are represented in Supp. Figure 7.

## DISCUSSION

In the reviewed literature, few articles have explored the use of MB in hemorrhagic shock in animal models. This study is the first to evaluate the early use of MB for treating hemorrhagic shock in a fixed-pressure pig model. In 2001, Jeroukhimov et al.^[5]^ compared prehospital hypotensive resuscitation protocols with volume resuscitation in a fixed-volume hemorrhage rat model, and evaluated the use of MB as an inhibitor of free radical formation. They concluded that MB could reduce the deleterious effects of volume resuscitation with electrolyte solutions alone and of ischemia and reperfusion injury. Ghiassi et al.^[6]^ evaluated the use of MB after the onset of refractory hemorrhagic shock in dogs and observed improvements in the clinical laboratory parameters of animals that received MB in combination with lactated Ringer's solution.

There are three experimental models of hemorrhagic shock: uncontrolled hemorrhage, fixed-volume, and fixed-pressure. The uncontrolled hemorrhage model simulates vascular trauma and, although it has more fidelity to real trauma, it is a model challenging to reproduce and compare. In fixed-volume hemorrhage models, the examiner determines the percentage of blood volume removed over a period. Its advantage is the study of physiological responses and natural compensatory mechanisms after losing a limited volume of blood; however, it is more difficult to standardize and reproduce because hypotension is not adequately defined^[7]^.

The fixed-pressure hemorrhage model, initially described by Penfield and later adopted by Widger, withdraws blood to maintain the recommended pressure^[8,9]^. The advantage of this method is the greater control of hypotension by monitoring blood pressure; thus, it is more standardized and reproducible, which is why it was used in this experiment. Despite this, it has the limitation of not reflecting real life because shock is maintained under constant pressure and may suffer from the effects of anesthesia and heparinization of the catheters used for blood withdrawal, significantly affecting the experimental results^[10]^.

The main conclusion of the present study was that the concomitant use of MB with BT allowed for the recovery of blood pressure levels, reaching higher levels than the use of blood replacement alone or the use of MB at different time from BT. It was also observed that the isolated use of MB did not have any effect on blood pressure; that is, its effect on pressure recovery depended on its association with volume, corroborating the observation of Ghiassi^[6]^ that the combination of MB with a volume-limited Ringer's lactate solution improved hemodynamic stability and reduced ischemic damage in resuscitation models of hemorrhagic shock in dogs. Notably, while Group 3 endured prolonged hypoperfusion, its recovery was similar to that of Group 4. This indicates that MB can assist in pressure recovery even when administered 60 minutes after the onset of shock, suggesting a potential window of opportunity while compensatory mechanisms are still effective. Further research is essential to identify the most advantageous timing for MB administration within the first hour of hemorrhagic shock to optimize therapeutic benefits.

These findings are consistent with several previous studies that evaluated the use of MB in refractory septic, anaphylactic, and vasoplegic shock treatments and showed its role as an adjuvant in restoring blood pressure^[11]^. MB reverses vascular hyperreactivity by inhibiting cGMP production. cGMP is a cellular signaling agent involved in smooth muscle relaxation, resulting in a decrease in peripheral vascular resistance^[6]^. Thus, MB inhibits peripheral vascular relaxation, acts as a vasoplegic inhibitor, and cannot be considered a vasopressor, as observed in the present study, owing to the lack of a pressure response to the use of the drug without its association with volume replacement.

One known adverse effect of MB is pulmonary hypertension, which occurs due to pulmonary vasoconstriction, resulting in decreased gas exchange and low oxygen saturation^[12]^. Regarding PAP, although all experimental animals presented high baseline levels of PAP values, all groups presented a similar pattern of PAP behavior. In the present study, there is no evidence that MB elevates pulmonary pressure.

In terms of PCP and CVP, the only notable difference was observed in Group 2, which did not receive blood and consequently maintained low values in these parameters. As for CI, vascular resistances, and their indexed values, no differences were noted among the groups. The potential absence of differences could be attributed to compensatory mechanisms and the relatively brief observation period.

For arterial blood acid-base, electrolyte, HB, Ht, and lactate levels, no response pattern was associated with the experimental groups. The nitrate dosage, an indirect method for measuring NO, showed no statistical differences between the experimental groups. Previous experiments have reported a similar response pattern when MB was used in anaphylactic shock models^[13]^. There were no statistically significant differences in MDA levels between the groups. The lack of changes in lactate, NO, and MDA levels may be related to the short observation period. Further studies with longer observation times are needed to evaluate the changes in their levels.

The data found in this study reinforce the adjuvant role of MB in the reversal of hemorrhagic shock. Knowledge of MB's safety and its mechanisms of action in reversing shock extrapolated from other shock modalities can pave the way for investigating its effects associated with other volume expanders, such as crystalloids. One example of its possible benefits is treating major burns in which the MB may have an important role, which may include reducing vascular permeability, the amount of blood loss, and the quantity of vasoactive amines for maintaining pressure, as well as favoring coagulation and protecting the microcirculation^[14,15]^.

The data from this study and previous studies call attention to a possible paradigm shift. Further experimental studies should be conducted to evaluate whether early use can prevent refractoriness in late stages of shock. Also, further studies should be conducted using clinical protocols.

### Limitations

Possible limitations of this study include the experimental shock model used, which, being isobaric, does not reflect reality; animal research is still shifting from the use of unrealistic pressure-controlled, volume-controlled hemorrhagic shock models or uncontrolled hemorrhagic shock outcome models. However, animal outcome models of combined trauma and shock are required, and a significant challenge is to find a clinically realistic long-term method in humans. In addition, there are limiting factors, such as anaesthesia, which can interfere with physiological responses, and the small number of animals used.

## CONCLUSION

In conclusion, the concurrent use of MB with BT allowed the reversal of hemorrhagic shock, achieving higher arterial pressure levels compared to either BT alone or the separate administration of MB and BT. No significant differences were observed in shock reversal when combining MB and BT at 15 or 60 minutes, and the treatment proved to be a potentially safe strategy. These findings reinforce the potential utility of MB as an adjunctive therapy in the early resuscitation phase of hemorrhagic shock, suggesting its value in clinical settings.
